# ZIKA virus elicits P53 activation and genotoxic stress in human neural progenitors similar to mutations involved in severe forms of genetic microcephaly and p53

**DOI:** 10.1038/cddis.2016.266

**Published:** 2016-10-27

**Authors:** Vincent El Ghouzzi, Federico T Bianchi, Ivan Molineris, Bryan C Mounce, Gaia E Berto, Malgorzata Rak, Sophie Lebon, Laetitia Aubry, Chiara Tocco, Marta Gai, Alessandra MA Chiotto, Francesco Sgrò, Gianmarco Pallavicini, Etienne Simon-Loriere, Sandrine Passemard, Marco Vignuzzi, Pierre Gressens, Ferdinando Di Cunto

**Affiliations:** 1 PROTECT, INSERM, Unversité Paris Diderot, Sorbonne Paris Cité, Paris, France; 2 Department of Molecular Biotechnology and Health Sciences, Molecular Biotechnology Centre, University of Turin, Turin, Italy; 3 Institut Pasteur, Centre National de la RechercheScientifique UMR 3569, Viral Populations and Pathogenesis Unit, Paris, France; 4 UEVE UMR 861, I-Stem, AFM, Evry, France; 5 Institut Pasteur, Functional Genetics of Infectious Diseases Unit, Paris 75724, France; 6 CNRS URA3012, Paris 75015, France; 7 Département de Génétique, Hôpital Robert Debré, Paris, France; 8 Center for Developing Brain, King' s College, London, UK; 9 Neuroscience Institute of Turin, Turin, Italy

## Abstract

Epidemiological evidence from the current outbreak of Zika virus (ZIKV) and recent studies in animal models indicate a strong causal link between ZIKV and microcephaly. ZIKV infection induces cell-cycle arrest and apoptosis in proliferating neural progenitors. However, the mechanisms leading to these phenotypes are still largely obscure. In this report, we explored the possible similarities between transcriptional responses induced by ZIKV in human neural progenitors and those elicited by three different genetic mutations leading to severe forms of microcephaly in mice. We found that the strongest similarity between all these conditions is the activation of common P53 downstream genes. In agreement with these observations, we report that ZIKV infection increases total P53 levels and nuclear accumulation, as well as P53 Ser15 phosphorylation, correlated with genotoxic stress and apoptosis induction. Interestingly, increased P53 activation and apoptosis are induced not only in cells expressing high levels of viral antigens but also in cells showing low or undetectable levels of the same proteins. These results indicate that P53 activation is an early and specific event in ZIKV-infected cells, which could result from cell-autonomous and/or non-cell-autonomous mechanisms. Moreover, we highlight a small group of P53 effector proteins that could act as critical mediators, not only in ZIKV-induced microcephaly but also in many genetic microcephaly syndromes.

The achievement of normal brain size and structure during development requires the generation and survival of an appropriate number of neurons. Genetic and environmental conditions that affect the normal expansion of neuronal progenitors, the timing of their differentiation and the survival of their progeny may severely reduce the final number of brain cells, resulting in microcephaly. Congenital microcephaly (CM) is a heterogeneous group of disorders characterized by reduced head circumference at birth, to at least 3 S.D. below the mean.^1, 2, 3, 4, 5, 6^ Although CM can be an isolated abnormality, compatible with normal intelligence, it often becomes clinically relevant because of its association with invalidating comorbidities such as severe intellectual disability, cerebral palsy, epilepsy or other syndromic features.^6^ CM can be the result of rare genetic disorders, mostly characterized by autosomal recessive inheritance.^4, 7^ Even more frequently, CM is produced by environmental factors such as hypoxia, drugs and alcohol exposure, or infectious agents such as Rubella, Toxoplasmosis or cytomegalovirus (CMV).^6^ Zika virus (ZIKV), a mosquito-borne flavivirus originally identified in Uganda in 1947,^8^ is the latest addition to the list of infectious agents that may lead to microcephaly. Since 2015, the spreading of ZIKV infection in Brazil and throughout Latin America has been associated with a sharp increase of the incidence of severe CM,^8, 9, 10^ leading to the declaration of a 'Global Emergency' by the World Health Organization. The possibility of vertical transmission from mother to fetus was first supported by detection of ZIKV in the amniotic fluid of two pregnant women whose fetuses had been diagnosed with microcephaly.^11^ ZIKV was then found in microcephalic fetal brain tissue, together with multifocal dystrophic lesions in the cortex and subcortical white matter.^9^ ZIKV efficiently infects human neural progenitors cells derived from induced pluripotent stem cells or from embryonic stem (ES) cells, reducing their proliferation and causing their death, as it has first been shown in 2D cultures^12^ and then in human brain organoids.^13, 14, 15^ Recently, studies in mouse models have demonstrated that infection of developing mouse brain by direct injection^16^ or by vertical transmission from the mother^17, 18, 19^ severely affects brain growth. Although the causal link between ZIKV infection and microcephaly is firmly established, it is still not clear why the developing brain is specifically vulnerable. ZIKV can infect different cell types, such as skin keratinocytes, fibroblasts and dendritic cells.^20, 21^ Viral entry can take place through different receptors, including DC-SIGN, Tyro3, TIM-1 and especially AXL.^20^ High levels of AXL are also expressed in the developing human cortex by radial glial cells, astrocytes, endothelial cells and microglia,^22^ providing a possible explanation of how ZIKV could reach the proliferating neural precursors. The cytopathic effect of ZIKV in infected skin cell is characterized by increased autophagosome formation, vacuolation and strong interferon response.^20^ Accordingly, over 80% of postnatal ZIKV cases are asymptomatic, whereas the remaining cases only exhibit mild fever, cutaneous rash and joint pain for a period of 7 days.^10, 12, 13, 14, 20^ In contrast, cultured neuronal progenitors^12, 13, 14, 15^ and developing human^9^ and mouse^17, 18, 19^ brains infected by ZIKV display massive alterations, with prominent apoptotic cell death. In ZIKV-infected organoids, many apoptotic cells are not immunostained by antibodies directed to viral antigens,^15^ suggesting the involvement of non-cell-autonomous mechanisms. ZIKV induces TLR3 overexpression in human neurospheres and organoids, and TLR3-competitive inhibitor attenuated organoid shrinkage.^13^ Nevertheless, the mechanisms of cell-cycle dysregulation and apoptosis induction by ZIKV in neural progenitors are still largely obscure. A distinct possibility to explain the specificity of these phenotypes is that ZIKV infection may directly or indirectly affect some of the molecular mechanisms that are also implicated in the pathogenesis of genetic CM.^4, 7, 23^ In this report, we explored this possibility, by comparing the gene expression profile of induced pluripotent stem cell (iPSC)-derived human neural progenitors cells (hNPCs) infected with ZIKV,^12^ with the profiles of developing neural tissues obtained from three different mouse models of severe genetic microcephaly. We found that P53 activation is a likely convergence point between the analyzed genetic models and ZIKV-infected cells. Therefore, we set out to experimentally test whether this event actually occurs and how it may be connected to apoptosis and genotoxic stress. The results of these experiments are described and discussed.

## Results

### Comparison of gene expression profiles between ZIKV-infected neural progenitors and mouse models of genetic microcephaly

Previous analysis of gene expression by RNA-sequencing (RNA-seq) of iPSC-derived hNPCs revealed that ZIKV infection significantly alters the expression of thousands genes.^12^ Among the downregulated genes, a strong prevalence of cell-cycle-related pathways was observed, which together with FACS analysis indicated that ZIKV arrests cell proliferation.^12^ On the other hand, Gene Ontology (GO) analysis of upregulated genes displayed enrichment of proteins involved in transcription, protein transport and catabolic processes, together with increased expression levels of genes involved in the regulation of apoptosis.^12^ However, considering the very high number of genes modulated by ZIKV infection (3434 upregulated and 3414 downregulated genes; Supplementary Table S1), it is difficult to understand which alterations could be causally linked to ZIKV-induced microcephaly and which may only represent bystander effects. To address whether the molecular events induced by ZIKV infection display significant similarities with genetic microcephaly, we set out to compare the expression profiles of ZIKV-infected hNPCs^12^ with the profiles of developing neural tissues obtained from three different mouse models of severe genetic microcephaly, that is, CitK−/− mice,^24^ Magoh+/− mice^25^ and conditional Elp3-knockout mice.^26^ CitK is a ubiquitous protein implicated in midbody maturation,^27, 28, 29^ whose inactivation leads to cytokinesis failure and massive apoptosis in the mammalian developing brain.^24, 30^ We obtained RNA-seq data from control and CitK−/− developing cerebellum (Bianchi *et al.*, Gene Expression Omnibus data set no. GSE83465), which is the neural tissue more severely affected by CitK loss.^24^ Magoh protein is one of the core components of the exon junction complex, implicated in nonsense-mediated decay pathway.^31^ Magoh haploinsufficiency leads to microcephaly,^25^ through prolonged mitosis of cortical neural progenitors, accumulation of DNA damage, premature differentiation and P53-dependent apoptosis.^25, 32^

**Figure 1:**
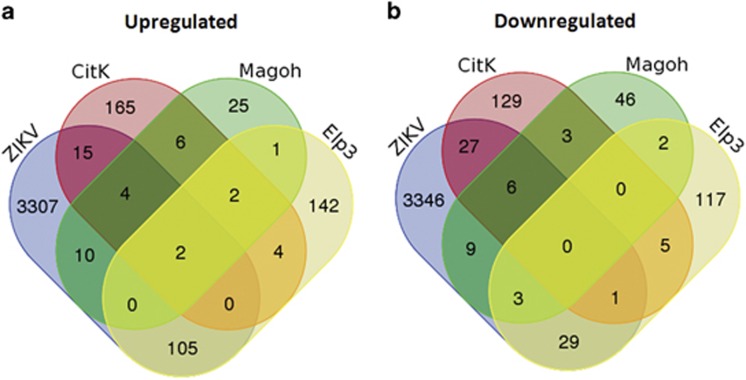
Comparison of gene expression profiles obtained from ZIKV-infected hNPCs and from mouse models of severe microcephaly. (**a**) Graphical representation of intersections between genes upregulated in ZIKV-infected hNPCs (ZIKV), CitK−/− P4 cerebellum (CitK), Magoh+/− E10.5 neocortex (Magoh) and Elp3−/− E13.5 brain (Elp3). The corresponding gene lists are reported in Supplementary Table S1. (**b**) Graphical representation of intersections between genes downregulated in the same conditions. Venn diagrams were obtained using the web tool http://bioinformatics.psb.ugent.be/webtools/Venn/

Elp3 is a catalytic histone acetyltransferase subunit of the RNA polymerase II elongator complex,^33^ which has been associated with transcriptional elongation and with specific modification of uridines at the wobble base position of tRNAs.^34^ Deletion of Elp3 results in impaired codon translation speed and triggers unfolded protein response (UPR), via activation of the PERK-eIF2*α*-Atf4 signaling.^26^ Conditional deletion of Elp3 in developing mouse brain leads to microcephaly, due to impairment of indirect neurogenesis, but not due to apoptosis.^26^ Gene expression profiles of E10.5 cortices obtained from Magoh+/− embryos and of E14.5 brains obtained from Elp3 conditional knockout mice were publicly available in the Gene Expression Omnibus (with accession numbers GSE19168 and GSE74683, respectively). In all these data sets, the number of significantly modulated genes is much lower compared with that in the ZIKV data set (Supplementary Table S1). Specifically, modulated genes were 370 in the CitK−/− study (199 upregulated and 171 downregulated), 119 in the Magoh+/− study (50 upregulated and 69 downregulated) and 413 in the Elp3 study (256 upregulated and 157 downregulated). Comparison of the genes in these lists (Figure 1) revealed a strong similarity between CitK and Magoh data sets, with 14 common upregulated genes (*P*=3.9E−17) and 9 common downregulated genes (*P*=6.9E−09). In addition, both data sets displayed a lower but statistically significant intersection with the Elp3 data set (Figure 1, *P*-value=3.1E−03 and 3.9E−04, respectively).

Considering also that CitK and Magoh data sets have been obtained on two different developing neural tissues (P4 cerebellum *versus* E10.5 cortex, respectively) and with two different gene expression analysis technologies (RNA-seq and DNA microarrays, respectively), these results are consistent with the higher phenotypical similarity between these two models of microcephaly, if compared with microcephaly resulting from conditional Elp3 deletion. Thus, the 14 upregulated genes and the 9 downregulated genes common to the CitK and to the Magoh data sets (Supplementary Table S2) are likely to have crucial roles in apoptotic microcephaly. Interestingly, 11 of the 14 common upregulated genes are P53 targets, and most of them have been involved in P53-dependent apoptosis and/or cell-cycle arrest (Supplementary Table S2). This result is consistent with the fact that both CitK−/− and Magoh+/− developing neural tissues display apoptosis.^24, 32^ Conversely, five of the nine common downregulated genes are well-known developmental regulators of the CNS (Supplementary Table S3). Notably, these genes include the transcription factor EOMES (TBR2), which is one of the most established markers for committed neural progenitors^35^ and may regulate the progression from neural stem cell to intermediate progenitors.^36^ Next, we compared the genes modulated in each microcephaly model with the genes showing altered expression in ZIKV-infected neural stem cells (Figure 1). CitK- and Magoh-upregulated genes showed a small but statistically significant overlap with ZIKV-upregulated genes (21 and 16 genes, *P*-values=2.7E−03 and 8E−04, respectively). Despite the limited overlap, six genes, corresponding to well-known targets of P53, were common to the three lists (expected 0.08 *P*<1E−10, *χ*^2^ test). Similarly, six genes were common to the corresponding lists of downregulated genes (expected 0.07 *P*<1E−10, *χ*^2^ test).

ZIKV-upregulated genes overlapped with Elp3-upregulated genes even more than with CitK- and Magoh-upregulated sequences (107 common genes *P*=9.5E−21; Supplementary Table S4), whereas the overlap between downregulated genes was not statistically significant. These common genes were enriched of aminoacyl-tRNA synthases (7E−8) by GO analysis.^37, 38^ Moreover, Gene Set Enrichment analysis (GSEA)^39, 40^ in the Molecular Signature Database (http://software.broadinstitute.org/gsea) revealed a strong overlap with genes upregulated in unfolded protein response (UPR) (set M5922, *P*=2.29E−9). Interestingly, ZIKV- and Elp3-upregulated genes include the P53 targets AEN, CCNG1, EDA2R and PHLDA3, which are shared with CitK- and Magoh-upregulated genes (Supplementary Table S2). Altogether, these observations indicate that ZIKV-infected cells and at least three independent mouse models of genetic microcephaly share the activation of P53-target genes.

### A signature of P53 activation is common to Zika and microcephaly models

To explore more, in general, the extent of P53 pathway activation in ZIKV-infected neural progenitors and in the analyzed microcephaly models, we resorted to GSEA, a powerful analytical method for interpreting gene expression data that focuses on specific gene sets obtained in different and heterogeneous studies.^40^ In particular, we concentrated our analysis on 50 gene expression signatures related to P53 function and evaluated their overlap with the lists of upregulated in the ZIKV, CitK, Magoh and Elp3 data sets. Strikingly, 32 of these data sets showed statistically significant overlap with ZIKV-upregulated genes (Table 1). As expected, a highly significant overlap was observed also with the CitK- and Magoh-upregulated genes, but the number of significant sets was lower compared with the ZIKV-upregulated genes (19 and 13 sets, respectively; Table 1). Interestingly, even Elp3-upregulated genes overlapped with many P53-related data sets (Table 1), further suggesting that P53 is activated in Elp3-deficient cortex (14 sets; Table 1). To evaluate whether P53 pathway activation is a specific feature of ZIKV-infected neural cells, rather than a nonspecific consequence of viral burden and highly deregulated transcriptome, we analyzed by GSEA the overlap between P53-related sets and genes upregulated in hNPCs after infection with the unrelated microcephaly virus CMV (Luo *et al.*,^41^ GEO accession number GSE19345). Strikingly, in this case only two sets showed significant overlap, and with relatively high *P*-values (Table 1). In addition, the overlap between genes upregulated by ZIKV and CMV was not significant. In contrast, we found a significant overlap between genes upregulated by CMV and downregulated by ZIKV (64 genes, *P*=6.10E−04; Supplementary Table S5). This group included many genes involved in cell-cycle control (Supplementary Table S5; *P*=4.1E−5), such as E2F1 and many of its targets. In conclusion, the above analysis of gene expression profiles revealed that, similarly to three microcephalic mutations, but differently from CMV, ZIKV activates in hNPCs an antiproliferative and proapoptotic P53-related response.

### P53 is activated in ZIKV-infected cells

**Figure 2:**
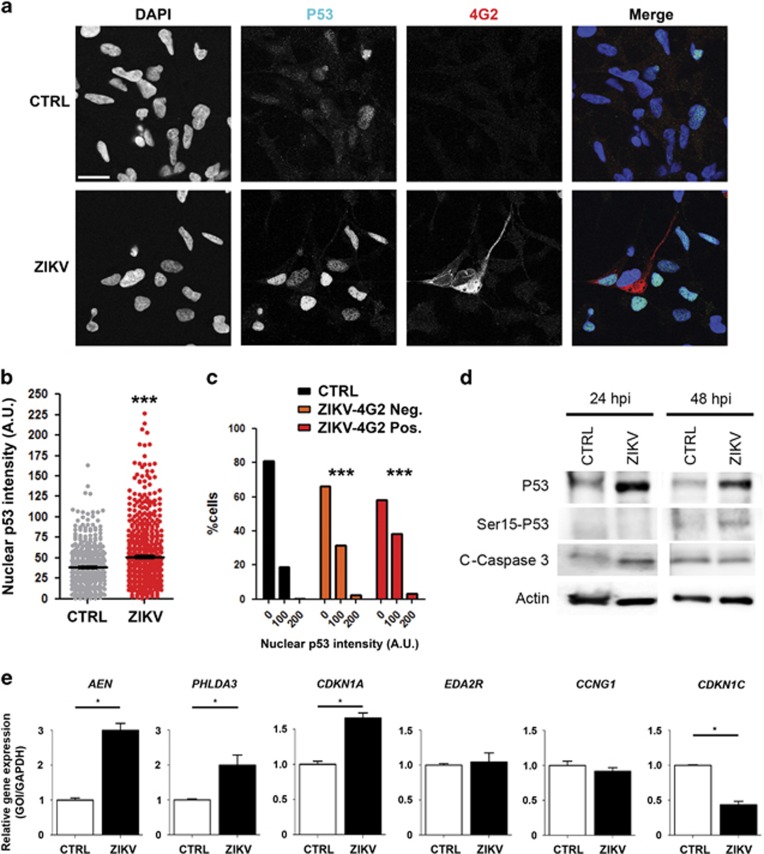
Increased P53 activation in ZIKV-infected hNPCs. (**a**) Control (CTRL) and infected (ZIKV) NSCi90 cells were analyzed 48 h after infection by immunofluorescence with anti-total P53 (P53) and anti-flavivirus antigens (4G2), and counterstained with DAPI (4',6-diamidino-2-phenylindole). The figure shows a representative high magnification field. Note the low or absent 4G2 signal in many of the infected cells showing strong P53 nuclear positivity. (**b**) Quantification of P53 nuclear intensity in arbitrary units (a.u.) in cells imaged in the above experiment (*n*=600 for each condition). (**c**) Distribution of P53 nuclear intensity in non-infected cells (CTRL) and in infected cells showing undetectable (ZIKV-4G2-Neg.) or detectable (ZIKV-4G2-Pos.) levels of flavivirus antigens. Quantifications in (**b**) and (**c**) are representative of three independent infections. Scale bar=10 *μ*m. (**d**) Total cell extracts of control cells and of cells infected with ZIKV for 24 or 48 h were analyzed by western blotting with the indicated antibodies. Actin, internal loading control; C-caspase, cleaved caspase. (**e**) Control or ZIKV-infected cells were analyzed by qRT-PCR for the indicated genes. GOI, gene of interest. Error bars, S.E.M. ***P<0.001; *P<0.05. Statistical significance was assessed by two-tailed unpaired Student's *t*-test in panels (**c** and **d**) and two-tailed Mann–Whitney *U*-test in panel (**e**)

The above data strongly suggest that P53 is activated by ZIKV infection, an event that could be critical for proliferative block and apoptosis induction. To validate this prediction, we performed P53 immunodetection in human NSCi90 hNPCs, under control conditions or after infection with the French Polynesian variant of ZIKV, which has been associated with microcephaly.^42, 43^ Increased nuclear accumulation of P53 is known to correlate with P53 activation.^44, 45^ ZIKV infection significantly enhanced the number of cells with P53-positive nuclei (Figures 2a and b), as well as the average nuclear P53 intensity (Figure 2b). Interestingly, counterstaining with antibodies directed against flavivirus proteins (4G2) did not show a good correlation between accumulation of viral antigens and P53 nuclear positivity (Figures 2a and c). Indeed, in the infected samples, the distribution of P53 nuclear intensity was essentially the same in 4G2-positive and -negative cells (Figure 2c). In agreement with the immunofluorescence results, western blotting analysis revealed a robust increase of P53 total protein levels, both 24 and 48 h after infection (Figure 2d). Moreover, blotting of the same protein extracts with antiphospho-Ser15 P53 antibodies revealed that this post-translational modification, an established marker of P53 functional activation,^46^ is significantly increased at 48 h (Figure 2d). To evaluate whether increased P53 levels and nuclear accumulation induced by ZIKV are accompanied by P53 functional activation, we analyzed by qPCR mRNA levels of five of the P53 targets reported in Supplementary Table S2. As expected, the expression of AEN, CDKN1A and PHLDA3 was significantly increased in the infected cells, whereas no differences were detected for EDA2R and CCNG1. Conversely, the expression of CDKN1C was significantly downregulated in the infected cells (Figure 2e), further validating our bioinformatic analysis.

### Apoptosis and genotoxic stress in ZIKV-infected cells

**Figure 3:**
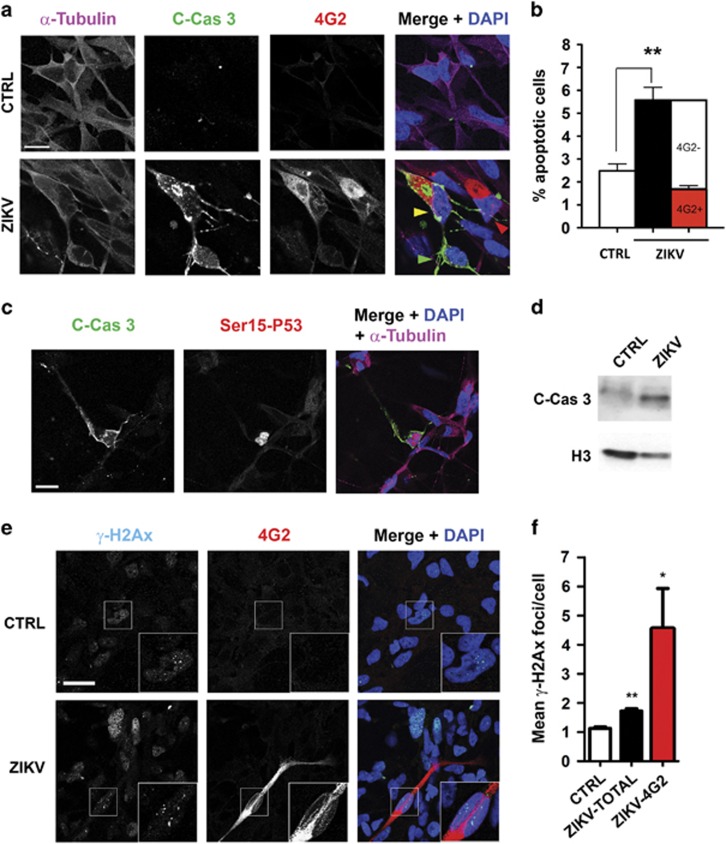
Increased P53 activation in ZIKV-infected hNPCs. (**a**) Control (CTRL) and infected (ZIKV) NSCi90 cells were analyzed 48 h after infection by immunofluorescence with anti-tubulin, anti-cleaved caspase-3 (C-cas 3) and anti-flavivirus antigens (4G2), and counterstained with DAPI (4',6-diamidino-2-phenylindole). The field shown in figure contains a caspase-positive/4G2-intense cell (yellow arrowhead in the merge), a caspase-positive cell with low 4G2 signal (green arrowhead) and a caspase-negative/4G2-positive cell (red arrowhead). Scale bar=5 *μ*m. (**b**) The first two bars of the histogram represent a quantification of the average frequency of caspase-3-positive cells in control and infected cells. The third bar represents the relative levels of 4G2-positive (red) and 4G2-negative (white) cells in caspase-3 positive cells of the infected samples. Error bars=S.E.M. (*n*=4000). (**c**) Representative image of ZIKV-infected cells stained with anti-cleaved caspase-3 and antiphospho-Ser15 P53. Positive nuclear stain with the latter antibody was observed in essentially all cells positive for cleaved caspase-3 (*n*=180). Scale bar=10 *μ*m. All data shown in the figure are representative of three independent infections. (**d**) Western blot analysis of cleaved caspase-3, normalized on histone H3. (**e**) IF analysis of *γ*H2Ax foci in control and ZIKV-infected cells 24 h after infection. Cells with multiple foci are magnified in the inset. Scale bar=10 *μ*m. (**f**) The average number of foci per nucleus was automatically quantified. ***P<0.001; **P<0.01 (two-tailed unpaired Student's *t*-test)

In agreement with previous reports, ZIKV-infected hNSC cultures displayed increased rate of apoptotic cells, as revealed by analysis of pyknotic nuclei (not shown) and by immunostaining with antibodies directed to activated caspase-3 (Figures 3a and b). Even in this case, no correlation was evident between apoptosis and viral cellular load in infected cultures, as ~60% of caspase-3-positive cells were negative for 4G2 antibody staining (Figure 3b). Most caspase-3-positive cells in ZIKV-infected cultures displayed increased levels of phospho-Ser15 P53 immunoreactivity, an established marker of active P53,^46^ which was especially concentrated in the nucleus (Figure 3c). Accordingly, western blotting analysis revealed increased caspase-3 levels (Figure 3d).

Many different viruses, including flaviviruses, are known to induce genotoxic stress in infected cells,^47, 48^ which is one of the main stimuli leading to P53 activation. Therefore, we assessed whether ZIKV-infected cells display increased DNA damage. To do so, we quantified nuclear foci of phosphorylated H2Ax histone (*γ*H2Ax), a well-established marker of DNA double-strand breaks.^49^ In comparison with other normal cell types, NSCi90 hNPCs cells display a relatively high average number of *γ*H2Ax foci already under basal conditions, consistent with the idea that neural progenitors may be particularly susceptible to replicative stress.^50^ Interestingly, 24 h after infection, ZIKV-infected cells displayed a significant increase in the average number of foci/nucleus, regardless of whether they showed detectable viral antigens or not (Figures 3d and e). However, the number of foci was consistently higher if only cells with visible 4G2 signal were considered (Figures 3d and e).

Altogether, our results demonstrate that cultures of hNPCs infected by ZIKV are characterized by genotoxic stress, P53 activation and apoptotic cell death and that the latter two events are not well correlated with the levels of 4G2 viral antigens.

## Discussion

In this report, we have provided bioinformatic evidence that transcriptional activation of P53 downstream effectors is the most consistent common molecular feature shared by ZIKV-infected hNPCs and by three different mouse models of microcephaly. Moreover and most importantly, we have shown that P53 is specifically activated by ZIKV infection in hNPCs. Previous studies in rodent models found that P53-induced apoptosis is responsible for neuronal loss in many different congenital microcephalies.^32, 51, 52, 53, 54, 55^ In some of these cases, P53 activation was correlated with increased DNA damage accumulation.^32, 52, 54, 55^ The recent report that DNA damage and consequent P53 activation can result from increased mitosis length in neural progenitors^32^ suggests that this mechanism could be much more common in microcephaly than previously thought, because many microcephaly genes have the potential to impact on mitosis timing.^4, 7^ However, P53-dependent apoptosis can also be produced through other mechanisms responsible for microcephaly syndromes, such as alteration of chromatin remodeling^56^ and block of miRNA function.^53, 57^ Our bioinformatic analysis further confirms and extends this concept, showing that a transcriptional signature of P53 activation characterizes not only the CitK and Magoh models but also the microcephaly produced by Elp3 deletion. The fact that in the latter case no apoptosis was detected^26^ is consistent with reports showing that UPR and ER stress, which characterizes Elp3 microcephaly,^26^ may activate mechanisms capable of preventing P53-dependent death.^58, 59^ Nevertheless, P53 activation and transcriptional induction of some P53 targets can still be sufficient to induce the growth arrest detected in Elp3-deficient neural progenitors.^26^

The high overlap between genes modulated by ZIKV infections and P53-related data sets (Table 1) suggested that ZIKV may strongly activate P53. Consistent with this prediction, we showed that ZIKV-infected hNPGs display increased P53 total levels, nuclear accumulation and Ser15 phosphorylation, which are all markers of P53 activation.^44, 45, 46^ Moreover, in ZIKV-infected cultures, most caspase-3-positive cells were positive for phospho-Ser15 P53, suggesting that P53 activation is responsible for apoptosis induction. Notably, P53 nuclear accumulation and caspase-3 positivity were detected not only in cells strongly positive for viral antigens but also in cells producing very low to undetectable levels of viral proteins (Figures 2 and 3). These results are consistent with those previously obtained in human brain organoids^15^ and in infected mouse brains,^16^ showing that apoptosis can be induced by ZIKV in cells apparently negative for virus expression. Altogether, they suggest that the cytopathic effects produced by ZIKV in developing neural tissue are not a simple consequence of cell stress deriving from viral burden, but may be the consequence of specific mechanisms leading to P53 activation. In support of this view, the response elicited on P53-related genes by CMV is quite at the opposite, with upregulation of proproliferative genes (Table 1 and Supplementary Table S4). Flaviviruses are known to activate P53 and to produce P53-dependent apoptosis.^60, 61^ In the case of West Nile Virus, it was shown that P53 activation may be caused by nucleolar sequestration of Hdm2 by capsid core protein.^61^ However, the P53 activation, which we detected in cells expressing low or undetectable levels of viral antigens, seems not compatible with this mechanism. A second possible cause of P53 activation is genotoxic stress, which could be produced by different mechanisms. For instance, ZIKV could induce the production of reactive oxygen species (ROS), as it has been shown in the case of hepatitis C virus (HCV).^62^ Moreover, decreased expression of DNA repair genes was detected in ZIKV-infected cells.^12^ We found that ZIKV infection increases DNA damage in cultured hNPCs (Figures 3d and e). This phenotype is already visible in cells with low to undetectable levels of viral antigens and become more pronounced with the increase of viral load (Figures 3d and e). Therefore, a likely possibility is that P53 activation by ZIKV is a cell-autonomous consequence of genotoxic stress. This scenario would imply that ZIKV can induce increased DNA damage very early during replication cycle, as the phenotype is already measurable when viral antigens are not yet clearly detectable by IF.

An alternative possibility to explain our results could be that DNA damage is not the cause, but rather a consequence of P53 activation. For instance, it could be the result of increased levels of P53-regulated nucleases, such as AEN. This scenario would be compatible with non-cell-autonomous mechanisms of P53 activation. In support of this view, it has been reported that ZIKV induces TLR3 overexpression and that TLR3 inhibition attenuates the effects of ZIKV on cell growth.^13^ In addition, studies in non-neural cells have shown that ZIKV induces interferon-*β* (IFN-*β*),^63^ which in turn may activate P53 and many proapoptotic P53-target genes, including TLR3.^64, 65, 66^ However, it must also be mentioned that IFN-β can promote survival of neural precursors,^67^ rather than killing them. In addition, it has been reported that ZIKV can have inhibitory roles on type-I IFN signaling, through STAT2 targeting.^68^

More studies are certainly necessary to dissect the cause-effect chain. However, as ZIKV can induce genotoxic stress, activate P53 and promote cell death when viral proteins are barely detectable (Figures 2 and 3), an outstanding question is how could infected cells become tolerant to high levels of DNA damage and high viral burden? To address this question, it may be helpful to consider that the gene expression profile of ZIKV-infected cells overlaps better with the Elp3 (characterized by ER stress but not apoptosis^26^) than with CitK and Magoh models (which display high apoptosis levels; Di Cunto *et al.*^24^ and Pilaz *et al.*^32^). As ER stress can protect cells from P53-dependent apoptosis,^58, 59^ it is possible that during the early stages of the infection cycle ZIKV-induced genotoxic stress may lead to a P53-dependent proapoptotic response. In the surviving cells, the accumulation of viral proteins could lead to UPR and ER stress, leading not only to cell adaptation and survival but also to cell-cycle arrest. A similar mechanism has already been demonstrated in the case of HCV, whose NS4B protein is capable not only of inducing a proapoptotic, ROS-dependent response but also of promoting a protective response mediated by the NF-*κ*B transcription factor.^62^ Interestingly, an independent bioinformatic analysis of the same data set considered in our study showed that NF-*κ*B pathway is activated in ZIKV-infected cells.^69^

In conclusion, although more investigations are required to discriminate between these possibilities, our study highlights P53 and a very limited number of P53-target genes as possible crucial mediators of ZIKV-induced growth arrest and apoptosis.

## Materials and Methods

### Comparison of expression profiles

RNA-seq data of ZIKV-infected hNPCs and controls were obtained from GEO RNA-seq data set GSE78711.^12^ The lists of differentially expressed genes (Supplementary Table S1) were defined as in the original paper.^12^

RNA-seq-based gene expression data of CitK−/− mice and controls were obtained from P4 developing cerebellum (Bianchi *et al.*, unpublished, data set is being submitted to GEO). Differentially expressed genes were defined using Cuffdiff v.2.0.2,^70^ with a 0.05 false discovery rate cutoff.

Microarray-based gene expression data of E10.5 Magoh-deficient mouse cortices^25^ were obtained from GEO data set GSE19168. The lists of differentially expressed genes (Supplementary Table S1) were defined as in the original paper.^25^

RNA-seq-based gene expression data of E13.5 Elp3−/− brains and controls^26^ were obtained from GEO data set GSE74683. The lists of differentially expressed genes (Supplementary Table S1) were defined as in the original paper.^26^

Microarray-based gene expression data of CMV-infected hNPCs^41^ were obtained from GEO data set GSE19345. Differentially expressed genes (Supplementary Table S1) were defined as those showing statistically significant deviation from control in the three time points of the time-course experiment, as defined the original paper.^41^

*P*-values of the intersections between the different lists were calculated using the Fisher's exact test. For GSEA analysis,^40^ P53-related data sets were downloaded from Molecular Signature Database (http://software.broadinstitute.org/gsea) and their overlap with genes upregulated in the above-described data sets was determined. *P*-values were then calculated using the Fisher's exact test, with Benjamini–Hochberg correction for 50 tests.

### Viral titration and infection

ZIKV strain PF13, isolated from French Polynesia, was amplified on Vero-E6 cells, maintained in DMEM containing 10% fetal calf serum (Sigma, St. Louis, MO, USA). For infection, virus was diluted in serum-free DMEM at the multiplicity of infection 1 (MOI 1). Viral inoculum was overlaid on cells for 30 min before medium replenishment. Supernatants were collected at indicated times for plaque assay where appropriate. For titration, dilutions of cell supernatant were prepared in serum-free DMEM and used to inoculate confluent monolayers of Vero-E6 cells for 30 min to 1 h at 37 °C. Cells were then overlaid with 0.8% agarose in DMEM containing 1.6% NBCS. ZIKV samples were incubated for 4 days. Following incubation, cells were fixed with 4% formalin and revealed with crystal violet solution (10% crystal violet (Sigma-Aldrich), 20% ethanol). Plaques were enumerated and used to back-calculate the number of plaque-forming units per milliliter of collected volume.

### Cell culture

Human i90c16 were derived from IMR-90 lung fibroblast cell (ATCC; Manassas, VA, USA, CCL-186) as described previously,^71^ using Addgene plasmid 20925, 20926 and 20927. Differentiation of i90c16 hiPSC into neural stem cells (NSCi90) was performed as described previously.^72, 73^

Cells were seeded on poly-ornithine/laminin- (Sigma) coated nitric acid-treated glass coverslips. The cells were grown in 50% DMEM:F12/50% Neurobasal medium (Invitrogen) supplemented with 1 × N2 (Invitrogen, Carlsbad, CA, USA), 1 × B27 (Invitrogen), 10 ng/ml FGF2 (Peprotech, Rocky Hill, NJ, USA) and EGF at 10 ng/ml (R&D Systems, Minneapolis, MN, USA). The medium was changed every alternate day and cells were passaged every 5 days. For infection with ZIKV, the medium was removed and virus was inoculated at MOI 1 in serum-free medium for 30 min. Wells were then replenished with fresh medium for 48 h.

### Immunocytochemical analysis

Control and infected NSCi90 were fixed for 20 min with 4% paraformaldehyde. Cells were permeabilized with 0.3% Triton 5 min X-100 for, washed, blocked for 1h with 10% serum in PBS and incubated with primary antibody overnight at 4 °C. Primary antibodies used were anti-p53 (7F5) (1:1600, no. 2527; Cell Signaling Technology, Boston, MA, USA), anti-D1-4G2-4-15 antibody (4G2) against flaviviruses (1:1500, MAB10216; Millipore, Darmstadt, Germany), antiphospho-Ser15 P53 (16G8) (1:400, no. 9286; Cell Signaling Technology), anti-activated caspase-3 (Asp175) (1:400, no. 9661; Cell Signaling Technology) and *γ*H2AX (S139, 20E3) (1:200, no. 9718; Cell Signaling Technology). Following washing, cells were treated with secondary antibody (1:1000 dilution) for 1h in dark at room temperature. Immunofluorescent cells were analyzed using a Leica TCS SP8 confocal scanning system (Leica Microsystems, Wetzlar, Germany) equipped with 488 nm Ar and 561 nm DPSS lasers. Eight-bit digital images were collected from a single optical plane using a × 40 HC PL APO CS2 oil-immersion Leica objective. For each optical section, double- or triple-fluorescence images were acquired in sequential mode to avoid potential contamination by linkage-specific fluorescence emission cross-talk. Settings for laser intensity, beam expander, pinhole (1 Airy unit), range property of emission window, electronic zoom, gain and offset of photomultiplicator, field format and scanning speed were optimized initially and held constant throughout the study so that all coverslips were digitized under the same conditions. Quantitative identification of ZIKV-positive and -negative cells, measurement of nuclear P53 signal intensity and quantification of the average number of *γ*H2AX foci were obtained using the Fiji Software.^74^ In particular, the latter quantification was obtained using the command 'Find Maxima' after the appropriate setting of the threshold and noise.

### Western blotting

Whole-cell extracts were fractionated by SDS-PAGE and transferred to a polyvinylidenedifluoride membrane. After incubation with either 10% BSA (P53 blot) or 10% nonfat milk (Ser15 P53, cleaved caspase-3 and actin blots) in TBST (10 mM Tris, pH 8.0, 150 mM NaCl, 0.5% Tween-20) for 1 h, membranes were incubated in 5% BSA or nonfat milk with antibodies against P53 (7F5, no. 2527; Cell Signaling Technology; dilution 1:1000), Ser15-phospho-P53 (16G8, no. 9286; Cell Signaling Technology; dilution 1:1000), cleaved caspase-3 (Asp175, no. 9661; Cell Signaling Technology; dilution 1:1000) or *β*-actin (MAB1501; Millipore; dilution 1:10000), histone H3 (1B1B2, no. 14269; Cell Signaling Technology; dilution 1:1000) overnight at 4 °C. Membranes were washed three times for 15 min and incubated for 1 h with horseradish peroxidase-conjugated anti-mouse or anti-rabbit antibodies. Blots were again washed three times with TBST and developed with the Western Lightning UltraRChemiluminescence Substrate (Perkin-Elmer Inc., Waltham, MA, USA) according to the manufacturer's protocols.

### Quantification of gene expression by real-time qPCR

Total RNA was extracted with the RNeasy Mini Kit (Qiagen, Hilden, Germany) according to the manufacturer's instructions. RNA quality and concentration were assessed by spectrophotometry with a Nanodrop apparatus (Thermo Fisher Scientific, Waltham, MA, USA). Total RNA (120 ng) was subjected to two independent reverse transcriptions using the iScript cDNA Synthesis Kit (Bio-Rad). qPCR was performed in duplicate on the 2 RT for each sample using SYBR Green Supermix (Bio-Rad) for 40 cycles with a two-step program (5 s of denaturation at 95 °C and 10 s of annealing at 60 °C) on a CFX384 (Bio-Rad, Marnes-la-Coquette, France). Amplification specificity was assessed with a melting-curve analysis. Primers were designed using the Primer3 plus Software (http://primer3plus.com). Specific mRNA levels were calculated after normalization of the results for each sample with those for GAPDH mRNA (reference gene). The data are presented as relative mRNA units with respect to control group (GOI/GAPDH expressed as fold over control value). Analyses were performed with the Bio-Rad CFX Manager 3.0 Software (http://primer3plus.com). Statistical analyses were performed using the GraphPadPrism 5.0 software (GraphPad Software, Inc., La Jolla, CA, USA) and results were compared using the Mann–Whitney *U*-test. A two-tailed *P*<0.05 was considered significant.

Primers used (5'–3') were as follows: hCDKN1C-FAGCTGCACTCGGGGATTT and hCDKN1C-RCTTCTCAGGCGCTGATCTCT; hEDA2R-FTGGATTGCCAAGAAAATGAG and hEDA2R-RGCATCTCCACCCTCTCCATA; hCCNG1-FTCCAAGCACAGAAGTGTGTAGAG and hCCNG1-RTGGTTTGGAACACTTATTTGATG; hAEN-FCATCACTCGGCAGCACAT and hAEN-RCCTGGAAGTCGTTGTGCAG; hCDKN1A-FGGAGACTCTCAGGGTCGAAA andhCDKN1A-RTAGGGCTTCCTCTTGGAGAA; hPHLDA3-FCAGGCCATCCAGACAGTG and hPHLDA3-RCCCCCACAAGCCAGAGG; hGAPDHAAAGGGTCATCATCTCTGCC and hGAPDHAGGGGTGCTAAGCAGTTGGT.

## Figures and Tables

**Table 1:** Genes upregulated in ZIKV-infected hNPCs and in mouse models of severe microcephaly significantly overlap with P53-related data sets

**MSigDB se**	**Zika**	**CMV**	**CitK−/−**	**Magoh+/−**	**Elp3−/−**
AMBROSINI_FLAVOPIRIDOL_TREATMENT_TP53	2.36E−06				
AMUNDSON_DNA_DAMAGE_RESPONSE_TP53	1.16E−06		2.95E−06	5.58E−06	5.57E−04
BIOCARTA_P53_PATHWAY	1.20E−03		6.20E−03	8.57E−04	
BIOCARTA_P53HYPOXIA_PATHWAY	1.50E−03			1.44E−03	
BRUINS_UVC_RESPONSE_VIA_TP53_GROUP_A	1.00E−35		5.29E−07		2.69E−04
BRUINS_UVC_RESPONSE_VIA_TP53_GROUP_B	5.65E−26	3.33E−03	9.11E−05	1.90E−04	1.23E−03
BRUINS_UVC_RESPONSE_VIA_TP53_GROUP_C	7.90E−03				
BRUINS_UVC_RESPONSE_VIA_TP53_GROUP_D	3.16E−13				
CEBALLOS_TARGETS_OF_TP53_AND_MYC_UP	9.92E−04				
HALLMARK_P53_PATHWAY	6.25E−34		1.45E−15	1.71E−16	1.62E−04
INGA_TP53_TARGETS	2.45E−04		3.49E−06	3.64E−08	
KANNAN_TP53_TARGETS_DN			9.50E−03		
KANNAN_TP53_TARGETS_UP	5.37E−08		6.10E−03		1.35E−03
KEGG_P53_SIGNALING_PATHWAY	7.91E−09		8.69E−09	2.15E−13	
MARTINEZ_RB1_AND_TP53_TARGETS_DN	1.48E−26		5.89E−08		8.07E−09
MARTINEZ_RB1_AND_TP53_TARGETS_UP	4.21E−21		2.07E−06		
MARTINEZ_TP53_TARGETS_DN	5.70E−26		7.78E−06		1.78E−07
MARTINEZ_TP53_TARGETS_UP	6.53E−23		1.27E−08		
MCMURRAY_TP53_HRAS_COOPERATION_RESPONSE_DN	1.47E−03				
ONGUSAHA_TP53_TARGETS	6.43E−07		1.94E−14	5.42E−11	
P53_DN.V1_DN	2.76E−04		1.05E−06	4.83E−03	2.73E−03
P53_DN.V1_UP	2.38E−10				2.44E−05
P53_DN.V2_DN	1.21E−03		1.28E−05		
PEREZ_TP53_AND_TP63_TARGETS	7.06E−18				1.60E−04
PEREZ_TP53_TARGETS	1.59E−50	7.54E−03	4.75E−04		1.17E−03
PID_P53_DOWNSTREAM_PATHWAY	5.42E−11		3.99E−10	4.07E−06	3.59E−03
PID_P53_REGULATION_PATHWAY	4.17E−11				
REACTOME_P53_DEPENDENT_G1_DNA_DAMAGE_RESPONSE	1.18E−03				
SCHAVOLT_TARGETS_OF_TP53_AND_TP63	1.63E−04		5.86E−03	7.62E−04	
SCIAN_INVERSED_TARGETS_OF_TP53_AND_TP73_DN	3.25E−07				
STAMBOLSKY_TARGETS_OF_MUTATED_TP53_UP	6.50E−07				
TANG_SENESCENCE_TP53_TARGETS_UP				2.70E−03	
V$P53_02	4.24E−22			9.72E−03	9.11E−03
V$P53_DECAMER_Q2	2.59E−13				
WU_APOPTOSIS_BY_CDKN1A_VIA_TP53					1.71E−04

Abbreviations: CMV, cytomegalovirus; GSEA, gene set enrichment analysis; hNPC, human neural progenitors cell; MSigDB, Molecular Signature Database; ZIKV, Zika virus

The table reports the significant (*P*<0.01) corrected *P*-values for the overlaps between the indicated GSEA data sets and the genes upregulated in ZIKV-infected hNPCs (ZIKV), CMV-infected hNPCs, CitK−/− P4 cerebellum, Magoh+/− E10.5 neocortex and Elp3−/− E13.5 brain. The corresponding gene lists are reported in Supplementary Table S1

## Abbreviations

ZIKV: Zika virus
CM: congenital microcephaly
CMV: cytomegalovirus
hNPC: human neural progenitors cell
iPSC: induced pluripotent stem cell
ES cells: embryonic stem
RNA-seq: RNA-sequencing
GO: gene ontology
GEO: Gene Expression Omnibus
UPR: unfolded protein response
GSEA: gene set enrichment analysis
MSigDB: Molecular Signature Database
ROS: reactive oxygen species
HCV: hepatitis C virus
