# Correction to: ZIKA virus elicits P53 activation and genotoxic stress in human neural progenitors similar to mutations involved in severe forms of genetic microcephaly

**DOI:** 10.1038/s41419-018-1159-8

**Published:** 2018-11-20

**Authors:** Vincent El Ghouzzi, Federico T. Bianchi, Ivan Molineris, Bryan C. Mounce, Gaia E. Berto, Malgorzata Rak, Sophie Lebon, Laetitia Aubry, Chiara Tocco, Marta Gai, Alessandra M. A. Chiotto, Francesco Sgrò, Gianmarco Pallavicini, Etienne Simon-Loriere, Sandrine Passemard, Marco Vignuzzi, Pierre Gressens, Ferdinando Di Cunto

**Affiliations:** 1 PROTECT, INSERM, Unversité Paris Diderot, Sorbonne Paris Cité, Paris, France; 2 Department of Molecular Biotechnology and Health Sciences, Molecular Biotechnology Centre, University of Turin, Turin, Italy; 3 Institut Pasteur, Centre National de la RechercheScientifique UMR 3569, Viral Populations and Pathogenesis Unit, Paris, France; 4 UEVE UMR 861, I-Stem, AFM, Evry, France; 5 Institut Pasteur, Functional Genetics of Infectious Diseases Unit, Paris, 75724 France; 6 CNRS URA3012, Paris, 75015 France; 7 Département de Génétique, Hôpital Robert Debré, Paris, France; 8 Center for Developing Brain, King’ s College, London, UK; 9 Neuroscience Institute of Turin, Turin, Italy

Correction to: *Cell Death and Disease* **7**, e2440 (2016); 10.1038/cddis.2016.266; published online 27Oct 2016.

The authors wish to point out that the name of the first author is appearing incorrectly on Pubmed: it should be El Ghouzzi V (and not Ghouzzi VE). In addition, the words “and p53” appear at the end of the title in the original publication (https://www.nature.com/articles/cddis2016266) and in the previous erratum version (https://www.nature.com/articles/cddis2016446). This is not correct.

