# Supplementary material for: ZIKA virus elicits P53 activation and genotoxic stress in human neural progenitors similar to mutations involved in severe forms of genetic microcephaly and p53
Source: Cell Death Dis. 2016 Oct 27;7(10):e2440–. doi: 10.1038/cddis.2016.266 (PMC5133962; doi:10.1038/cddis.2016.266)
Supplement: Supplementary Information [file cddis2016266x1.pdf]

Supplementary tables

Table S2

List of the genes upregulated in both CitK-/- and Magoh +/- developing neural tissue.

Gene Name	Gene ID	Implicated as p53 target gene	Implicated in cell cycle and mitosis control	Implicated in survival or apoptosis	UP in ZIKV	UP in Ulp3 KO
AEN	64782	(Kawase et al., 2008)		(Kawase et al., 2008)	+	+
AK1	203	(Collavin et al., 1999)	(Collavin et al., 1999;Pollock et al., 2014)	(Pollock et al., 2014)		
ANO3	63982					
ARAP2	116984					
CCNG1	900	(Okamoto and Beach, 1994)	(Smith et al., 1997)	(Russell et al., 2012)		+
CDKN1A	1026	(Dulic et al., 1994)	(Dulic et al., 1994)	(Dulic et al., 1994)	+	
DDIT4L	115265	(Ellisen et al., 2002)		(Imen et al., 2009)		
EDA2R	60401	(Tanikawa et al., 2009)		(Tanikawa et al., 2009)		+
PHLDA3	23612	(Kawase et al., 2009)		(Kawase et al., 2009)	+	+
PSRC1	84722	(Hsieh et al., 2002)	(Jang et al., 2011)	(Sun et al., 2008)		
SERPINE2	5270			(Boulaftali et al., 2013)		
SESN2	83667	(Budanov and Karin, 2008)	(Budanov and Karin, 2008)	(Ben-Sahra et al., 2013; Ding et al., 2015)	+	
TP53INP1	94241	(Imen et al., 2009)	(Okamura et al., 2001; Tomasini et al., 2005)	(Imen et al., 2009; Tomasini et al., 2005)	+	
ZMAT3	64393	(Varmeh-Ziaie et al., 1997)	(Hellborg et al., 2001)	(Higashi et al., 2002)	+	

Table S3

List of the genes downregulated in both CitK-/- and Magoh +/- developing neural tissue

Gene Name	Gene ID	Transcription regulator activity	Developmental protein	Neuron development	DOWN in ZIKV
CDKN1C	1028	+	+	+	+
DCC	1630	+	+	+	
DCX	1641		+	+	
EOMES	8320	+	+	+	+
IGFBPL1	347252				+
MYT1	4661	+	+	+	
NEUROG2	63973	+	+	+	+
ST18	9705	+			+
TAGLN3	29114				+

Table S4

List of the genes upregulated in both ZIKV-infected neural stem cells and Elp3 -/- developing neural tissue.

Gene Name	Gene ID	Aminoacyl-tRNA synthesis (GO)	Unfolded protein response (GSEA M5922)
AARS	16	+	
AEN	64782		
AKAP9	10142		
ALDH1L2	160428		
ANKRD11	29123		
ANKRD12	23253		
ARID4B	51742		
ASNS	440		+
ATF4	468		+
ATF5	22809		
BCAT1	586		
BDP1	55814		
BOD1L1	259282		
CARS	833	+	
CCAR1	55749		
CCDC112	153733		
CCDC174	51244		
CDK11A	728642		
CDK11B	984		
CEBPZ	10153		
CELF4	56853		
CEP290	80184		
CNR1	1268		
DBT	1629		
DMRT3	58524		
DNAJC2	27000		
DNAJC21	134218		
DPF2	5977		
DUSP4	1846		
EIF2S2	8894		
EIF4EBP1	1978		+
ELAVL3	1995		
ESCO1	114799		
FAT3	120114		
FUBP3	8939		
GARS	2617	+	
GCC2	9648		
GPATCH1	55094		
GPT2	84706		
GRIA2	2891		
IARS	3376	+	+

IRS1	3667		
ITSN2	50618		
KIAA2026	158358		
KIF5B	3799		+
KTN1	3895		
MARS	4141	+	
MPHOSPH10	10199		
MTHFD1L	25902		
MTHFD2	10797		+
MTSS1	9788		
NARS	4677	+	
NCOR1	9611		
NEMF	9147		
NFIA	4774		
NIPBL	25836		
NKAP	79576		
NOP58	51602		
NRP2	8828		
NRXN1	9378		
PCDH19	57526		
PCF11	51585		
PHGDH	26227		
PHLDA3	23612		
PLCB1	23236		
PPIL4	85313		
PPP1R12A	4659		
PPP1R15B	84919		
PPP3CA	5530		
PRPF38B	55119		
PSAT1	29968		+
PSPH	5723		
PYCR1	5831		
PYGO1	26108		
R3HDM1	23518		
RB1CC1	9821		
RBBP6	5930		
RBM25	58517		
RBM26	64062		
RBM39	9584		
RFX4	5992		
ROCK1	6093		
ROCK2	9475		
RSBN1L	222194		
RSRC2	65117		
SALL1	6299		
SCAPER	49855		
SEC62	7095		
SHMT2	6472		
SLC3A2	6520		

**SLC6A9**	6536		
**SLC7A5**	8140		+
**SMC3**	9126		
**SOX9**	6662		
**SPEN**	23013		
**TAF1D**	79101		
**TARS**	6897	+	+
**TMF1**	7110		
**TOP1**	7150		
**TRIP11**	9321		
**TRIP6**	7205		
**UNC5B**	219699		
**YARS**	8565	+	
**ZC3H15**	55854		
**ZFP37**	7539		
**ZNF292**	23036		
**ZNF788**	388507		

Table S5

List of the genes downregulated by ZIKV and upregulated by CMV in infected human neural progenitor cells.

Gene Name	Gene ID	Cell cycle/ Mitosis
AGPAT2	10555	
AGPAT3	56894	
ARNTL2	56938	
AURKA	6790	+
C11orf75	56935	
C1orf115	79762	
C7orf25	79020	
CALML4	91860	
CCNE1	898	+
CDC25A	993	+
CDCA8	55143	+
CDKN3	1033	+
CDT1	81620	+
CENPE	1062	+
CNNM3	26505	
COL12A1	1303	
CTSL2	1515	
DEPDC1	55635	
E2F1	1869	+
E2F2	1870	+
ENPP1	5167	
EPB41L2	2037	
EPHB2	2048	
FANCA	2175	+
FKBP5	2289	
FOXRED2	80020	
H2AFY	9555	
KCNK1	3775	
KPNB1	3837	
LDLRAP1	26119	
MCM10	55388	+
MCM5	4174	+
MKI67	4288	+
MSRB3	253827	
NEFH	4744	
NHLH1	4807	

**NMU**	10874	
**NT5DC3**	51559	
**ORAOV1**	220064	
**PASK**	23178	
**PENK**	5179	
**PLAGL1**	5325	+
**PLS1**	5357	
**RAD51**	5888	+
**RAD54L**	8438	+
**RASL10B**	91608	
**RHBDL3**	162494	
**RRM2**	6241	+
**SFXN2**	118980	
**SHMT1**	6470	
**SLC39A8**	64116	
**SLC43A3**	29015	
**SLC45A4**	57210	
**SLCO4A1**	28231	
**SPTLC2**	9517	
**TCF19**	6941	
**TFDP1**	7027	+
**TK1**	7083	+
**TMEM140**	55281	
**TMEM164**	84187	
**TSPAN33**	340348	
**UBE2T**	29089	
**VANGL1**	81839	
**ZWINT**	11130	+

Supplementary references

Ben-Sahra, I., Dirat, B., Laurent, K., Puissant, A., Auberger, P., Budanov, A., Tanti, J. F., and Bost, F. (2013). Sestrin2 integrates Akt and mTOR signaling to protect cells against energetic stress-induced death. Cell Death Differ *20*, 611-619.

Boulaftali, Y., Francois, D., Venisse, L., Jandrot-Perrus, M., Arocas, V., and Bouton, M. C. (2013). Endothelial protease nexin-1 is a novel regulator of A disintegrin and metalloproteinase 17 maturation and endothelial protein C receptor shedding via furin inhibition. Arterioscler Thromb Vasc Biol *33*, 1647-1654.

Budanov, A. V., and Karin, M. (2008). p53 target genes sestrin1 and sestrin2 connect genotoxic stress and mTOR signaling. Cell *134*, 451-460.

Collavin, L., Lazarevic, D., Utrera, R., Marzinotto, S., Monte, M., and Schneider, C. (1999). wt p53 dependent expression of a membrane-associated isoform of adenylate kinase. Oncogene *18*, 5879-5888.

Ding, B., Parmigiani, A., Yang, C., and Budanov, A. V. (2015). Sestrin2 facilitates death receptor-induced apoptosis in lung adenocarcinoma cells through regulation of XIAP degradation. Cell Cycle *14*, 3231-3241.

Dulic, V., Kaufmann, W. K., Wilson, S. J., Tlsty, T. D., Lees, E., Harper, J. W., Elledge, S. J., and Reed, S. I. (1994). p53-dependent inhibition of cyclin-dependent kinase activities in human fibroblasts during radiation-induced G1 arrest. Cell *76*, 1013-1023.

Ellisen, L. W., Ramsayer, K. D., Johannessen, C. M., Yang, A., Beppu, H., Minda, K., Oliner, J. D., McKeon, F., and Haber, D. A. (2002). REDD1, a developmentally regulated transcriptional target of p63 and p53, links p63 to regulation of reactive oxygen species. Mol Cell *10*, 995-1005.

Hellborg, F., Qian, W., Mendez-Vidal, C., Asker, C., Kost-Alimova, M., Wilhelm, M., Imreh, S., and Wiman, K. G. (2001). Human wig-1, a p53 target gene that encodes a growth inhibitory zinc finger protein. Oncogene *20*, 5466-5474.

Higashi, Y., Asanuma, M., Miyazaki, I., Haque, M. E., Fujita, N., Tanaka, K., and Ogawa, N. (2002). The p53-activated gene, PAG608, requires a zinc finger domain for nuclear localization and oxidative stress-induced apoptosis. J Biol Chem *277*, 42224-42232.

Hsieh, S. C., Lo, P. K., and Wang, F. F. (2002). Mouse DDA3 gene is a direct transcriptional target of p53 and p73. Oncogene *21*, 3050-3057.

Imen, J. S., Billiet, L., Cuaz-Perolin, C., Michaud, N., and Rouis, M. (2009). The regulated in development and DNA damage response 2 (REDD2) gene mediates human monocyte cell death through a reduction in thioredoxin-1 expression. Free Radic Biol Med *46*, 1404-1410.

Jang, C. Y., Coppinger, J. A., Yates, J. R., 3rd, and Fang, G. (2011). Mitotic kinases regulate MT-polymerizing/MT-bundling activity of DDA3. Biochem Biophys Res Commun *408*, 174-179.

Kawase, T., Ichikawa, H., Ohta, T., Nozaki, N., Tashiro, F., Ohki, R., and Taya, Y. (2008). p53 target gene AEN is a nuclear exonuclease required for p53-dependent apoptosis. Oncogene *27*, 3797-3810.

Kawase, T., Ohki, R., Shibata, T., Tsutsumi, S., Kamimura, N., Inazawa, J., Ohta, T., Ichikawa, H., Aburatani, H., Tashiro, F., and Taya, Y. (2009). PH domain-only protein PHLDA3 is a p53-regulated repressor of Akt. Cell *136*, 535-550.

Okamoto, K., and Beach, D. (1994). Cyclin G is a transcriptional target of the p53 tumor suppressor protein. Embo J *13*, 4816-4822.

Okamura, S., Arakawa, H., Tanaka, T., Nakanishi, H., Ng, C. C., Taya, Y., Monden, M., and Nakamura, Y. (2001). p53DINP1, a p53-inducible gene, regulates p53-dependent apoptosis. Mol Cell *8*, 85-94.

Pollock, A., Bian, S., Zhang, C., Chen, Z., and Sun, T. (2014). Growth of the developing cerebral cortex is controlled by microRNA-7 through the p53 pathway. Cell Rep *7*, 1184-1196.

Russell, P., Hennessy, B. T., Li, J., Carey, M. S., Bast, R. C., Freeman, T., and Venkitaraman, A. R. (2012). Cyclin G1 regulates the outcome of taxane-induced mitotic checkpoint arrest. Oncogene *31*, 2450-2460.

Smith, M. L., Kontny, H. U., Bortnick, R., and Fornace, A. J., Jr. (1997). The p53-regulated cyclin G gene promotes cell growth: p53 downstream effectors cyclin G and Gadd45 exert different effects on cisplatin chemosensitivity. Exp Cell Res *230*, 61-68.

Sun, W. T., Hsieh, P. C., Chiang, M. L., Wang, M. C., and Wang, F. F. (2008). p53 target DDA3 binds ASPP2 and inhibits its stimulation on p53-mediated BAX activation. Biochem Biophys Res Commun *376*, 395-398.

Tanikawa, C., Furukawa, Y., Yoshida, N., Arakawa, H., Nakamura, Y., and Matsuda, K. (2009). XEDAR as a putative colorectal tumor suppressor that mediates p53-regulated anoikis pathway. Oncogene *28*, 3081-3092.

Tomasini, R., Seux, M., Nowak, J., Bontemps, C., Carrier, A., Dagorn, J. C., Pebusque, M. J., Iovanna, J. L., and Dusetti, N. J. (2005). TP53INP1 is a novel p73 target gene that induces cell cycle arrest and cell death by modulating p73 transcriptional activity. Oncogene *24*, 8093-8104.

Varmeh-Ziaie, S., Okan, I., Wang, Y., Magnusson, K. P., Warthoe, P., Strauss, M., and Wiman, K. G. (1997). Wig-1, a new p53-induced gene encoding a zinc finger protein. Oncogene *15*, 2699-2704.
